# *Ccl21a*, Rather Than *Ccl21b*, is Essential for Thymocyte Migration in Mouse

**DOI:** 10.1002/eji.70114

**Published:** 2025-12-20

**Authors:** Izumi Ohigashi, Hitomi Kyuma, Eri Otsu, Shinichi Hayashi, Tatsuya Takemoto, Yousuke Takahama

**Affiliations:** ^1^ Division of Experimental Immunology Institute of Advanced Medical Sciences Tokushima University Tokushima Japan; ^2^ Laboratory of Developmental Immunology Institute of Photonics and Human Health Frontier Tokushima University Tokushima Japan; ^3^ Laboratory For Embryology Institute of Advanced Medical Sciences Tokushima University Tokushima Japan; ^4^ Faculty of Medicine Department of Anatomy Kansai Medical University Osaka Japan; ^5^ Thymus Biology Section, Experimental Immunology Branch National Cancer Institute, National Institutes of Health Bethesda Maryland USA

**Keywords:** CCL21, gene duplication, medullary thymic epithelial cell, thymus

## Abstract

Self‐tolerance in T cells is a vital self‐defense strategy for mammals to specifically respond to invading pathogens. During T cell development in the thymus, thymocytes migrate from the cortex to the medulla to sequentially acquire non‐self‐reactivity and self‐tolerance. This cortex‐to‐medulla migration is regulated by CCR7‐mediated chemokine signaling. Previous studies have identified CCL21 but not CCL19 as a functional ligand for this CCR7‐dependent migration. CCL21 in the mouse is encoded by multiple genes, including CCL21Ser‐encoding *Ccl21a* and several CCL21Leu‐encoding genes, including *Ccl21b*. The importance of *Ccl21a* in thymocyte migration has been demonstrated, whereas the role of CCL21Leu‐encoding genes remains unclear. By producing mice specifically deficient in *Ccl21b*, we show that *Ccl21b* plays little to no role in the cortex‐to‐medulla migration of developing thymocytes. CCL21Leu‐encoding gene transcripts remain detectable even in the absence of *Ccl21b*, suggesting that *Ccl21b* is not a major source of CCL21Leu. We further show that the copy number of CCL21Leu‐encoding genes is smaller than the currently estimated copy number in a public database. These findings underscore the predominant role of *Ccl21a* over *Ccl21b* in the mouse thymus.

## Graphical abstract

CCR7‐mediated chemokine signaling regulates the medullary migration of thymocytes to establish self‐tolerance. By comparing the thymic phenotype of *Ccl21b*‐KO mice with that of *Ccl21a*‐KO mice, we show that *Ccl21a* plays a predominant role compared with *Ccl21b* among CCR7 ligands in the thymus.

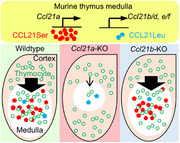

## 1 Introduction

**FIGURE 1:**
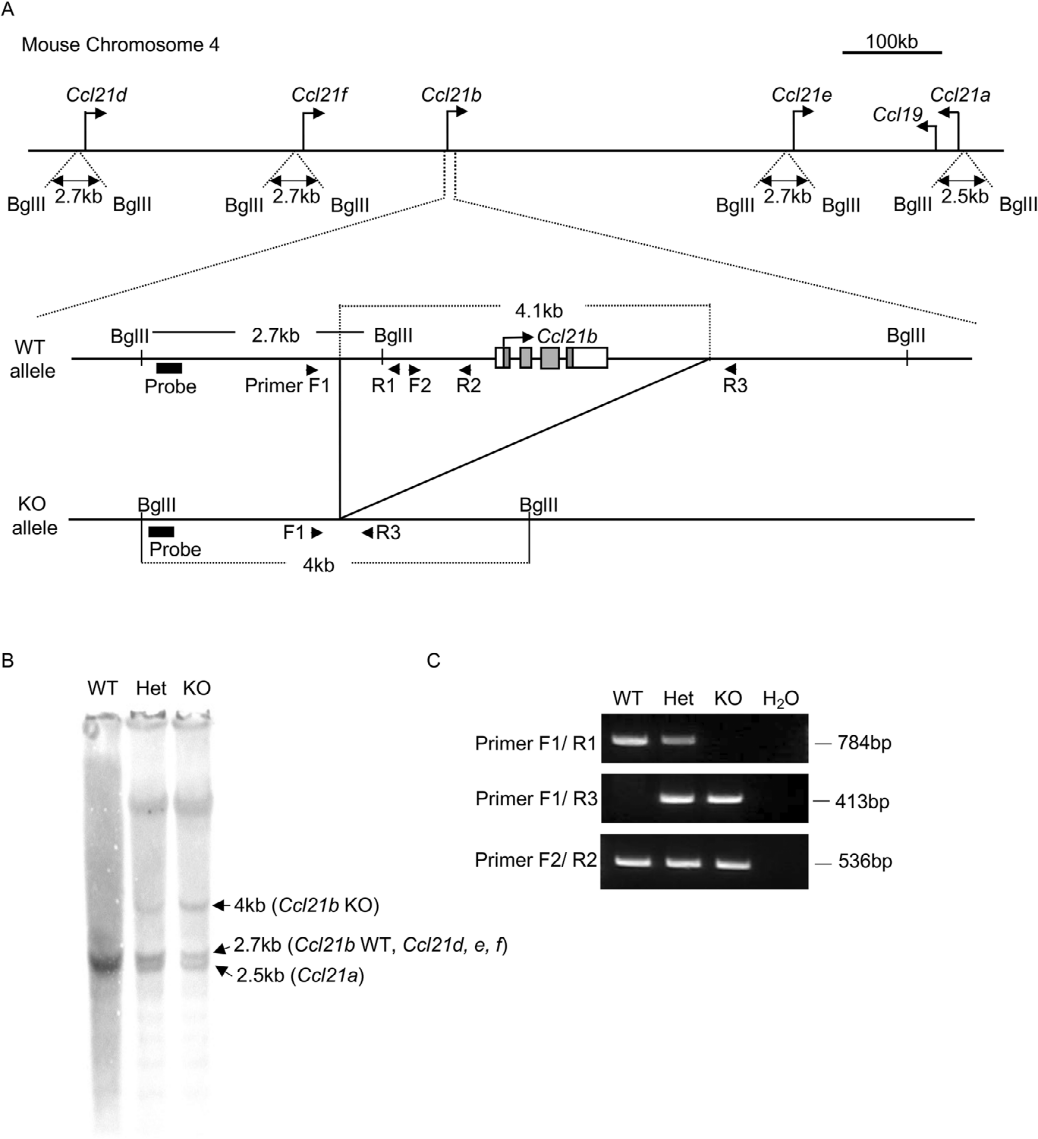
Engineering of *Ccl21b*‐KO mice. (A) Schematic diagram of *Ccl21* genomic loci on mouse chromosome 4. Wildtype (WT) and knockout (KO) alleles of *Ccl21b* are also shown. The probe is for Southern blot analysis. Arrowheads indicate PCR primers. (B) Southern blot analysis of BglII‐digested genomic DNA from indicated mice. The probe is shown in (A). (C) Gel electrophoresis image of genomic PCR analysis. Primer positions are shown in (A).

During T cell development in the thymus, the migration of positively selected cortical thymocytes to the medulla is crucial for the establishment of self‐tolerance in T cells by inducing the negative selection of T cells with self‐reactivity and promoting regulatory T cell generation [1, 2]. The cortex‐to‐medulla migration of developing thymocytes is predominantly regulated by C–C chemokine receptor 7 (CCR7)‐mediated chemokine signaling in which medullary thymic epithelial cells (mTECs) provide CCR7 ligands [3, 4, 5, 6, 7]. CCL19 and CCL21 are ligands of CCR7, and CCL21 in mice is encoded by multiple genes [8, 9, 10, 11]. CCL21Ser, which has a serine at the 65th amino acid position, is encoded by *Ccl21a*. CCL21Leu, which has a leucine at the 65th position and is one amino acid different from CCL21Ser, is encoded by four copies of the genes *Ccl21b*, *Ccl21d*, *Ccl21e*, and *Ccl21f*, according to the public database provided by the National Center for Biotechnology Information (NCBI) (Figure 1A).

Previous reports have demonstrated that mice deficient in *Ccl21a* exhibit impaired cortex‐to‐medulla migration of developing thymocytes and defective medullary deletion of self‐reactive thymocytes, consequently leading to the development of autoimmune dacryoadenitis, whereas *Ccl19*‐knockout (KO) mice do not [12, 13, 14]. These findings have highlighted the importance of CCL21Ser but not CCL19 in the medullary migration of thymocytes. However, the in vivo role of CCL21Leu‐encoding genes remains unclear.

In this study, we generated mice in which one of the CCL21Leu‐encoding genes was deleted. We focused on *Ccl21b* because it is best known as a CCL21Leu‐encoding gene [10, 11]. We herein report that, unlike *Ccl21a*‐KO mice, *Ccl21b*‐KO mice exhibit an undiminished expression of CCL21 protein in the thymic medulla and an undisturbed cortex‐to‐medulla migration of developing thymocytes. We also report that the estimated copy number of CCL21Leu‐encoding genes in the mouse genome is two, smaller than the currently estimated four in the NCBI database. These results underscore the importance of *Ccl21a* and CCL21Ser in regulating thymocyte traffic in the mouse thymus.

## 2 Results and Discussion

### 2.1 Generation of *Ccl21b*‐KO Mice

To examine the role of *Ccl21b* *in vivo*, we generated mice specifically deficient in *Ccl21b* by CRISPR/Cas9‐mediated genome editing, in which a 4.1 kb genomic region containing *Ccl21b* was deleted (Figure 1A). Southern blot analysis of BglII‐digested genomic DNA revealed the appearance of a *Ccl21b*‐KO‐specific 4 kb fragment in KO DNAs, indicating successful deletion of the *Ccl21b* sequence (Figure 1B). Because the sequences for all *Ccl21* loci were highly homologous (Figure S1A), the same probe also detected a 2.5 kb fragment containing *Ccl21a* and a 2.7 kb wild‐type (WT) fragment containing other CCL21Leu‐encoding genes, including *Ccl21d*, *Ccl21e*, and *Ccl21f*, in accordance with the NCBI database, in the genomic DNA of *Ccl21b*‐KO mice (Figure 1A,B). The deletion of *Ccl21b* was further confirmed through the amplification of a 413 bp genomic fragment using primers F1 and R3, whereas the 784 bp WT fragment amplified with primers F1 and R1 was lost (Figure 1C). In contrast, a 536 bp WT fragment amplified with primers F2 and R2 did not disappear in the genomic DNA of *Ccl21b*‐KO mice (Figure 1C). This is due to the high homology in the F2/R2 region among *Ccl21b*, *Ccl21d*, *Ccl21e*, and *Ccl21f* loci, because we found that the sequence amplified from *Ccl21b*‐KO genomic DNA was identical to the sequences in *Ccl21e* and *Ccl21f* loci but distinct from the sequence in *Ccl21b* or *Ccl21d* locus (Figure S1B).

### 2.2 Quantification of CCL21Leu‐Encoding Genes

**FIGURE 2:**
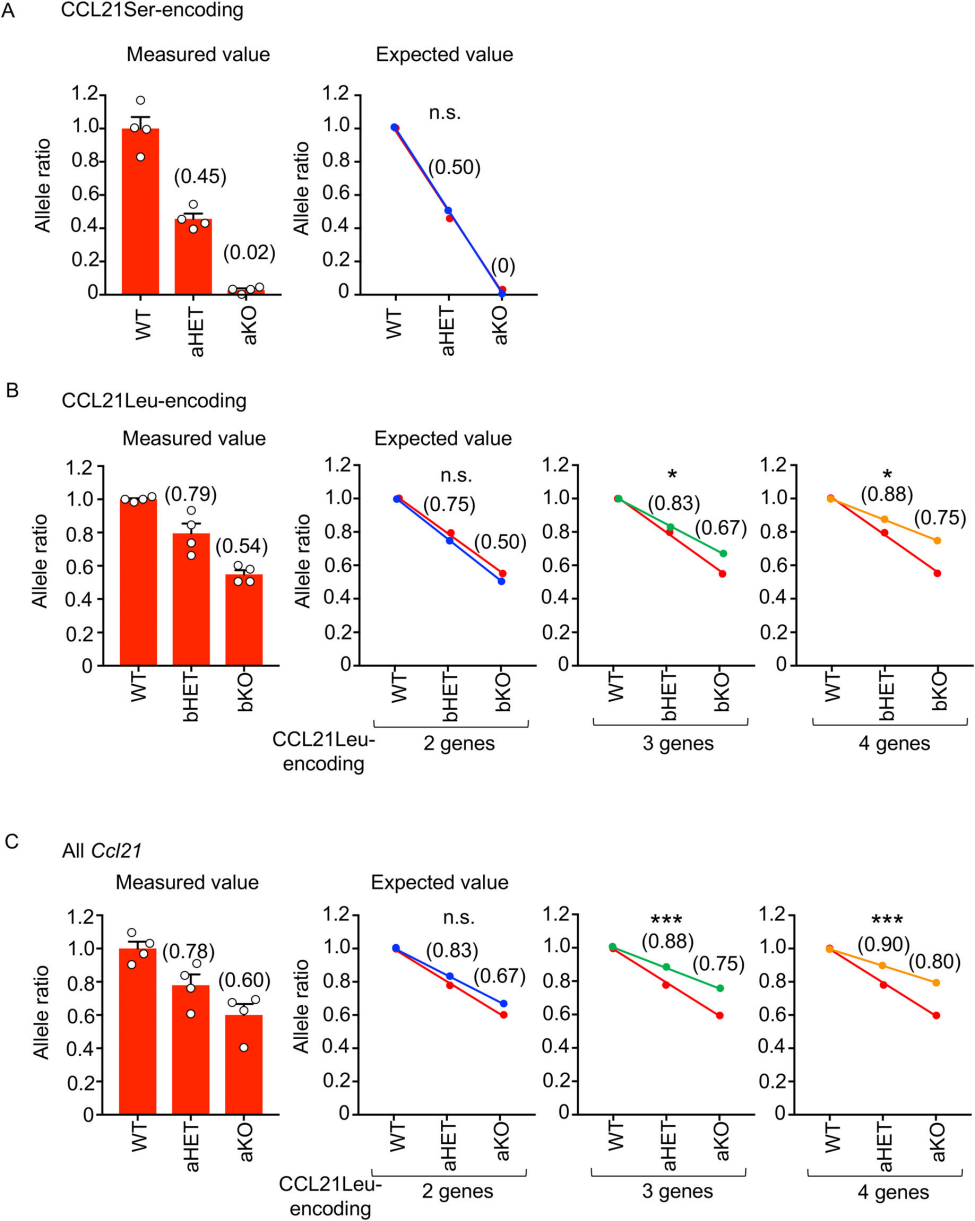
Quantification of *Ccl21* genes in the mouse genome. Quantitative PCR analysis of (A) CCL21Ser‐encoding *Ccl21a* , (B) CCL21Leu‐encoding genes, and (C) all *Ccl21* genes in genomic DNA from indicated mice. WT, wild type; aHET, *Ccl21a*‐heterozygous KO mice; aKO, *Ccl21a*‐homozygous KO mice; bHET, *Ccl21b*‐heterozygous KO mice; bKO, *Ccl21b*‐homozygous KO mice. The bar graphs on the left side of each panel show allele ratios (means and SEs; *n* = 4) measured by quantitative genomic PCR. Numbers in parentheses represent mean values of the detected allele ratios. The plots and the numbers in parentheses in the remaining graphs in each panel indicate the expected allele ratios calculated from the allele numbers. Red lines indicate the regression of measured allele ratios. Blue, green, and yellow lines represent the expected allele ratios from the estimated copy numbers. Comparisons of slopes between measured values and expected values were performed using analysis of covariance. *p < 0.05; ***p < 0.001; n.s., not significant.

Southern blot analysis of genomic DNA demonstrated that the signal intensity of the 2.7 kb fragment was comparable to, and not apparently less robust than, that of the 4 kb KO fragment in *Ccl21b*‐KO mice (Figure 1B), contradicting the possible existence of three additional copies of CCL21Leu‐encoding genes (*Ccl21d*, *Ccl21e*, *Ccl21f*) in the mouse genome. Consequently, we examined the DNA amount of CCL21Leu‐encoding genes through quantitative genomic PCR analysis. Control analysis of CCL21Ser‐encoding *Ccl21a* in the genomic DNAs revealed a linear loss of *Ccl21a*, namely, halved in *Ccl21a*‐heterozygous (HET) mice and completely lost in *Ccl21a*‐homozygous KO mice, in agreement with the notion that there is only one *Ccl21a* locus in the mouse genome (Figure 2A). In contrast, genomic amplification utilizing primers that detected CCL21Leu‐encoding genes *Ccl21b*, *Ccl21d*, *Ccl21e*, and *Ccl21f* demonstrated that the loss of *Ccl21b* in *Ccl21b*‐homozygous KO mice reduced the signals by 50%, indicating that in addition to *Ccl21b*, there exists one more CCL21Leu‐encoding gene in the B6 mouse genome (Figure 2B). Accordingly, a parallel analysis of all *Ccl21* genes, that is, *Ccl21a*, *Ccl21b*, *Ccl21d*, *Ccl21e*, and *Ccl21f*, in the genomic DNA showed that the loss of *Ccl21a* left the signals for two additional genes, reconfirming that there are two copies of CCL21Leu‐encoding genes in the B6 mouse genome (Figure 2C). These results indicate that only two CCL21Leu‐encoding genes are present in the B6 mouse genome and that the copy number of CCL21Leu‐encoding genes is overestimated in the NCBI database. Among the CCL21Leu‐encoding genes, 10 kb‐spanning genomic sequences between *Ccl21e* and *Ccl21f* have the highest homology (99.9%), followed by those between *Ccl21b* and *Ccl21d* (99.7%) (Figure S1A). Indeed, sequencing analysis detected the presence of *Ccl21e* and/or *Ccl21f* sequences (Figure S1B). Consequently, we consider it likely that *Ccl21b* and either *Ccl21e* or *Ccl21f* are present in the B6 mouse genome to encode CCL21Leu, rather than four copies of CCL21Leu‐encoding *Ccl21b*, *Ccl21d*, *Ccl21e*, and *Ccl21f* loci as annotated in the NCBI database.

### 2.3 Predominant Role of *Ccl21a* Over *Ccl21b* in the Thymus

**FIGURE 3:**
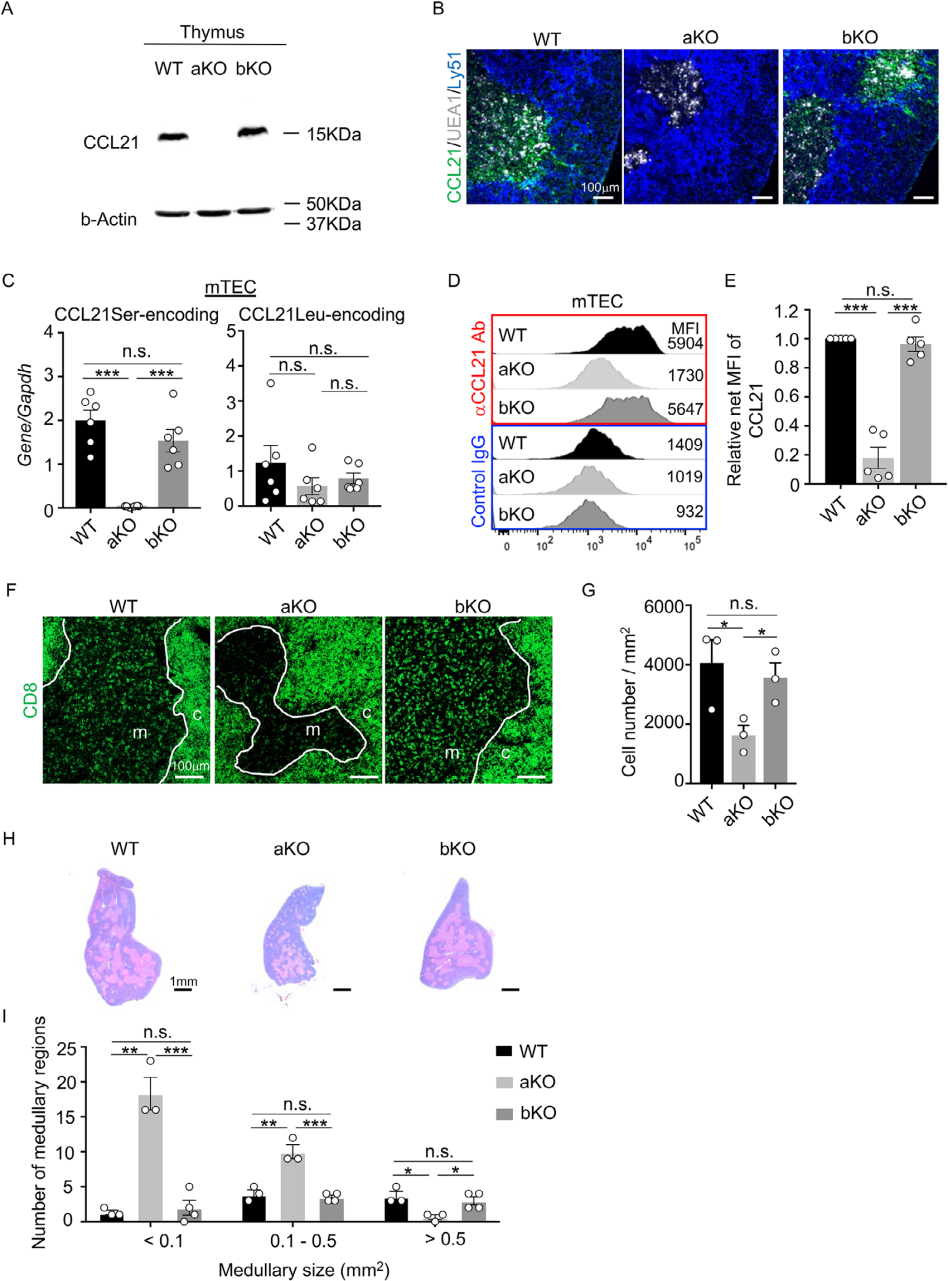
Predominant role of *Ccl21a* over *Ccl21b* in the thymus (A) Immunoblot analysis of CCL21 protein in the thymus isolated from WT, aKO (*Ccl21a*‐homozygous KO), and bKO (*Ccl21b*‐homozygous KO) mice. β‐Actin was examined as loading control. (B) Immunofluorescence analysis of CCL21 (green), UEA1 (gray), and Ly51 (blue) in the thymus of 7‐week‐old mice. (C) Quantitative RT‐PCR analysis of CCL21Ser‐ and CCL21Leu‐encoding genes (means and SEs; *n* = 6) in CD45^−^EpCAM^+^UEA1^+^Ly51^−^ mTECs isolated from indicated mice. (D) Flow cytometric profiles of anti‐CCL21 antibody and control IgG staining in CD45^−^EpCAM^+^UEA1^+^Ly51^−^ mTECs from the thymus of 7‐week‐old mice. Numbers in the profiles indicate median fluorescence intensity. (E) Relative net fluorescence intensity (means and SEs; *n* = 5) of CCL21 in mTECs of indicated mice. Median fluorescence intensity of CCL21in WT mTECs was set as 1. (F, G) Medullary accumulation of thymocytes. (F) Immunofluorescence analysis of CD8 (green) in thymic sections of 7‐week‐old mice. Bars, 100 µm. c, cortex. m, medulla. (G) Means and SEs (*n* = 3) of CD4^−^CD8^+^ thymocyte numbers per unit area (mm^2^) in the medullary region. (H) Hematoxylin and eosin staining of thymic sections of 7‐week‐old mice. Bar, 1 mm. (I) Number of medullary regions of a given size in the largest coronal thymic sections of indicated mice. The *x*‐axis indicates medullary size (mm^2^), and the *y*‐axis indicates the number of medullary regions (means and SEs; *n* = 3). All images and the histogram are representative results from at least three independent experiments. *p < 0.05; **p < 0.01; ***p < 0.001; n.s., not significant.

We then analyzed the thymus from *Ccl21b*‐KO and *Ccl21a*‐KO mice. In agreement with our previous report [12], immunoblot and immunofluorescence analyses using an antibody that recognized both CCL21Ser and CCL21Leu proteins (Figure S2A) revealed that CCL21 protein in the thymus of *Ccl21a*‐KO mice was essentially undetectable, whereas CCL21 protein in the thymus of *Ccl21b*‐KO mice was readily detectable in the medullary region, similar to that in the thymus of WT mice (Figure 3A,B). Quantitative RT‐PCR analysis of CCL21Ser‐ and CCL21Leu‐encoding genes using specific primers (Figure S2B) indicated that *Ccl21a* expression was severely reduced in mTECs isolated from *Ccl21a*‐KO mice, but not in mTECs isolated from WT or *Ccl21b*‐KO mice (Figure 3C). In contrast, CCL21Leu‐encoding gene expression was comparable among mTECs isolated from WT, *Ccl21a*‐KO, and *Ccl21b*‐KO mice (Figure 3C), suggesting that *Ccl21b* is not a predominant source of CCL21Leu. Flow cytometric analysis further demonstrated that CCL21 protein detected in mTECs was comparable between WT and *Ccl21b*‐KO mice but was remarkably diminished in *Ccl21a*‐KO mice (Figure 3D,E). Nonetheless, the faint CCL21 signal detected in *Ccl21a*‐KO mTECs (Figure 3D,E) might reflect a trace amount of CCL21Leu protein in the absence of CCL21Ser. However, it is also possible that nonspecific binding by an antibody reagent caused the faint fluorescence signal. We also found that medullary accumulation of developing thymocytes was comparable between WT and *Ccl21b*‐KO mice, but was significantly reduced in *Ccl21a*‐KO mice (Figure 3F,G). Mutual interaction between medullary thymocytes and mTECs is essential for the development of the thymic medulla [15, 16, 17, 18]. Reduced interaction due to impaired thymocyte migration resulted in defective medullary formation, characterized by an increase in small fragmental medullary regions and a decrease in large medullary regions in *Ccl21a‐KO* mice (Figure 3H,I) [12]. However, the distribution of small and large medullary regions was comparable between WT and *Ccl21b*‐KO mice (Figure 3H,I). Furthermore, we found that the CD4/CD8 profiles of thymocytes and the cellularity of thymocyte subsets, including those in CD4^−^CD8^−^ cells defined by CD44 and CD25 expression, were comparable between *Ccl21b*‐KO mice and WT mice, as in *Ccl21a*‐KO mice (Figure S3). These results indicate that *Ccl21b* is dispensable for the production of CCL21 protein in mTECs and the medullary accumulation of thymocytes, highlighting the importance of *Ccl21a* over *Ccl21b* in the mouse thymus. Considering that the amount of CCL21 protein was markedly reduced in the *Ccl21a*‐KO thymus (Figure 3A,B), and that the copy number of *Ccl21a* is higher than that of CCL21Leu‐encoding *Ccl21* in mTECs [12], we think CCL21Ser is the predominant form in the thymus, even in the *Ccl21b*‐KO thymus.

It was reported that CCL21Leu‐encoding gene transcripts were predominantly detected in nonlymphoid organs, including the lung [11, 12, 19]. However, similarly to the expression in mTECs, CCL21Leu‐encoding gene expression in the lung was not reduced by the lack of *Ccl21b*, whereas *Ccl21a* expression was impaired in the lung of *Ccl21a*‐KO mice (Figure S4A). The expression of CCL21Leu‐encoding genes was slightly, although not significantly, reduced in both lung cells and mTECs from *Ccl21a*‐KO mice (Figure 3C; Figure S4A), possibly due in part to the long‐range positional effect of the overall *Ccl21* locus modification in these mice. Importantly, immunoblot analysis revealed a marked reduction in CCL21 protein levels in the lung of *Ccl21a*‐KO mice but not *Ccl21b*‐KO mice (Figure S4B), indicating that CCL21Ser is substantially expressed in the lung.

The numbers of chemokines and chemokine receptors vary among species [20]. This variation can be explained by the birth‐and‐death model, in which new genes are generated through duplication. While some duplicated genes are retained in the genome for extended periods, others are inactivated or deleted during the evolutionary process [20, 21]. Because CCL21Leu is unable to compensate for the loss of CCL21Ser in the mouse thymus, the genes encoding CCL21Leu may represent evolutionary remnants from past chemokine gene diversification. Alternatively, CCL21Leu may have an unexpected role in nonlymphoid organs or may function synergistically with CCL21Ser. We attempted to generate *Ccl21a/Ccl21b* double‐KO mice by intercrossing those two single‐KO strains. However, because the two loci are in proximity on the same chromosome, we were unable to generate those mice. To clarify the functional significance of CCL21Leu *in vivo*, it will be useful to examine mice deficient in all CCL21Leu‐encoding genes.

## 3 Concluding Remarks

The present study highlights the significant role of *Ccl21a* compared with *Ccl21b* in the thymus. We have recently reported that *Ccl21a*‐expressing mTECs in the fetal thymus exhibit progenitor activity, giving rise to Aire‐expressing mTECs [22] that present tissue‐restricted self‐antigens necessary for the acquisition of T cell self‐tolerance [23, 24]. Therefore, *Ccl21a*‐expressing mTECs are essential not only for the migration of thymocytes into the medulla but also for the generation of diverse mTEC subsets. Elucidating the mechanisms underlying the roles of *Ccl21a* and *Ccl21b* in the thymus, including those regulating the expression of CCL21Ser‐encoding and CCL21Leu‐encoding genes in mTECs, will enhance our understanding of central tolerance mechanisms in the adaptive immune system.

## 4 Materials and Methods

### 4.1 Mice

C57BL/6 (B6) mice were obtained from SLC Japan. *Ccl21a*‐KO mice were described previously [12]. Mice were housed in a climate‐controlled, pathogen‐free barrier facility on a 12‐hour light‐dark cycle. All mouse experiments were performed using age‐ and sex‐matched mice.

### 4.2 Generation of *Ccl21b*‐KO Mice

*Ccl21b*‐KO mice were generated as previously described [25]. Briefly, zygotes from C57BL/6 mice were electroporated with Cas9 protein (IDT), tracrRNA (IDT), and two crRNAs flanking *Ccl21b*, *Ccl21b* crRNA1: tagaacttgctctatagagcagg, and *Ccl21b* crRNA2: ctgtgcacatctcagtgtgtagg. The electroporated embryos that developed into the 2‐cell stage were transferred into the oviduct of pseudopregnant female mice. The primers used for PCR genotyping are shown in Table S1.

### 4.3 Southern Blotting

Genomic DNA extracted from the liver was digested with BglII, electrophoresed in 1% agarose, and transferred to a nylon membrane (GE Healthcare). The probe was labeled with a PCR DIG Probe Synthesis Kit (Roche), and hybridization was detected using anti‐DIG‐AP Fab fragment (Roche), CDP‐STAR (Roche), and Light Capture II (Atto).

### 4.4 Quantitative PCR Analysis

The primer sequences used for quantitative genomic and reverse transcription‐PCR analyses are shown in Table S1. For quantitative genomic PCR analysis, genomic DNA was extracted from mouse ears, and 3 ng of DNA was used for amplification. Each cycle threshold (Ct) value was normalized to the Ct value of *Rag2*. For quantitative reverse transcription‐PCR analysis, total cellular RNA was reverse‐transcribed with a PrimeScript Reverse Transcription Kit (Takara). Quantitative real‐time PCR was performed using SYBR Premix ExTaq (Takara) and a StepOnePlus Real‐Time PCR System (Applied Biosystems). The amplified products were confirmed to be single bands by gel electrophoresis.

### 4.5 Western Blotting

Cell lysates in lysis buffer (150 mM NaCl, 1% NP‐40, 0.5% deoxycholate, 0.1% SDS, 50 mM Tris‐HCl, pH 8.0) supplemented with the protease inhibitor cocktail (Sigma) were subjected to SDS‐PAGE, transferred onto a polyvinylidene difluoride membrane (Millipore), and probed with antibodies listed in Table S2. Signals were detected with ECL reagent (GE Healthcare) and LAS‐4000mini (FUJIFILM).

### 4.6 Immunofluorescence Analysis

Paraformaldehyde‐fixed and frozen thymuses embedded in OCT compound (Sakura Finetek) were sliced into 10‐µm‐thick sections. The sections were stained with antibodies listed in Table S2. Images were analyzed under a TCS SP8 confocal laser scanning microscope (Leica).

### 4.7 Hematoxylin and Eosin Staining

Paraformaldehyde‐fixed and frozen sections were stained with hematoxylin and eosin (Muto Pure Chemicals Co.) and observed under an Eclipse E1000 microscope (Nikon). The size (mm^2^) of medullary regions in the thymic sections was measured by using Photoshop software (Adobe).

### 4.8 Flow Cytometric Analysis and Cell Sorting

For the analysis of TECs, minced thymuses were digested with 0.5 U/mL Liberase (Roche) in the presence of 0.02% DNase I (Roche). Single‐cell suspensions of the thymus were stained with antibodies specific for EpCAM, CD45, and Ly51, and for reactivity with UEA‐1 (Table S2). For the analysis of CCL21, surface‐stained cells were fixed in 4% (g/vol) paraformaldehyde, permeabilized using a Foxp3 Staining Buffer Set (Invitrogen), and stained with anti‐CCL21 antibody. For the analysis of thymocytes, cells were stained with the indicated antibodies. Then, the antibody‐stained cells were analyzed using FACSVerse (BD). For the isolation of mTECs and lung cells, CD45^−^ cells were enriched in magnetic bead‐conjugated anti‐CD45 antibody (Miltenyi Biotec). The CD45^−^‐enriched thymic cells were cell‐surface stained and sorted by FACSAria II (BD).

### 4.9 Statistical Analysis

Data were analyzed using one‐way ANOVA with Tukey's correction. Comparisons of the slopes of the regression lines in Figure 2 were performed using analysis of covariance.

### 4.10 Constructs and Transfection

*Ccl21a* and *Ccl21b* complementary DNAs were PCR‐amplified from B6 mice and *Ccl21a*‐KO mTECs, respectively, using PrimeSTAR DNA polymerase (TaKaRa). The amplified products were cloned into a pCR‐blunt vector (Invitrogen) and subsequently subcloned into a CMV‐promoter‐driven mammalian expression plasmid. HEK293T cells were cultured in Dulbecco's Modified Eagle Medium supplied with 10% fetal bovine serum and 100 U/mL penicillin–streptomycin at 37°C and 5% CO_2_. Cells were transfected using X‐tremeGENE 9 DNA Transfection Reagent (Roche).

## Author Contributions

Izumi Ohigashi designed and performed the experiments, analyzed and interpreted the data, and wrote the manuscript. Hitomi Kyuma and Eri Otsu performed the experiments. Shinichi Hayashi and Tatsuya Takemoto generated the gene‐edited mice. Yousuke Takahama interpreted the data and wrote the manuscript.

## Ethics Statement

All mouse experiments were approved by the Animal Experimentation Committee of Tokushima University (T2022‐50 and T2022‐107).

## Conflicts of Interest

The authors declare no conflicts of interest.

## Supporting information




**Supporting File**: eji70114‐sup‐0001‐SuppMat.pdf.

## Data Availability

The data that support the findings of this study are available from the corresponding author upon reasonable request.
