# Supplementary material for: Ccl21a, Rather Than Ccl21b, is Essential for Thymocyte Migration in Mouse
Source: Eur J Immunol. 2025 Dec 20;55(12):e70114. doi: 10.1002/eji.70114 (PMC12717634; doi:10.1002/eji.70114)
Supplement: Supplementary file 1 — Supporting File: eji70114‐sup‐0001‐SuppMat.pdf. [file EJI-55-e70114-s001.pdf]

Supporting information

1. Supplemental Figures S1–S4
2. Supplemental Tables S1 and S2
3. Western blot raw data

A

	Ccl21a	*Ccl21b*	*Ccl21d*	*Ccl21e*	*Ccl21f*
Ccl21a					
Ccl21b	94.0%				
Ccl21d	93.7%	99.7%			
Ccl21e	97.1%	98.0%	97.9%		
Ccl21f	97.1%	98.0%	97.9%	99.9%	

B

Ccl21b/d AGACAGTGATAACAACGTA

Ccl21e/f AGACAGTGATAACGATGTA

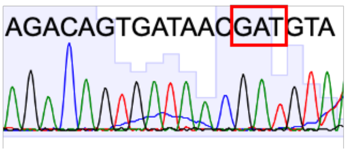

Supplementary Figure S1. Genomic sequences of CCL21-encoding gene loci

(A) Sequence homology of 10 kb genomic regions spanning from 5 kb upstream to 5 kb downstream of the indicated CCL21-encoding genes. Genomic DNA sequences were obtained from the NCBI database (https://www.ncbi.nlm.nih.gov/).

(B) Comparison of genomic sequences within the F2/R2 region of *Ccl21b/d* and *Ccl21e/f* loci shown in Figure 1A (upper). Sequence within the 536 bp fragment amplified from *Ccl21b*-KO genomic DNA using F2 and R2 primers (bottom). Red boxes indicate a mismatch between *Ccl21b/d* and *Ccl21e/f* sequences.

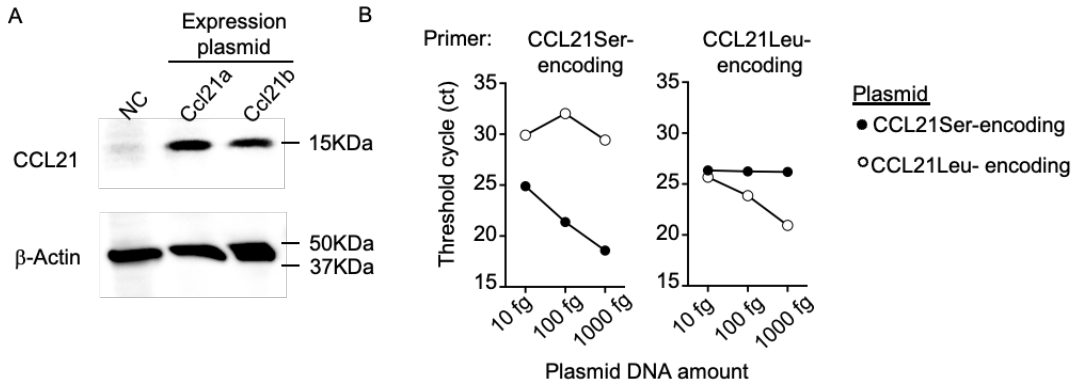

Supplementary Figure S2. Specific detection of CCL21Ser- and CCL21Leu-encoding genes

(A) Immunoblot analysis of CCL21 protein in HEK293T cells transfected with either *Ccl21a* or *Ccl21b* expression plasmid. β-Actin signals were examined as loading control. NC, negative control cells not transfected with the expression plasmid.

(B) Graphs show threshold cycle (ct) values obtained by quantitative PCR of plasmid DNAs containing CCL21Ser-encoding *Ccl21a* (filled circles) and CCL21Leu-encoding genes (open circles), amplified using primers specific for the CCL21Ser-encoding gene (left) and the CCL21Leu-encoding genes (right). The x-axis indicates the amount of plasmid DNA used in the PCR amplification.

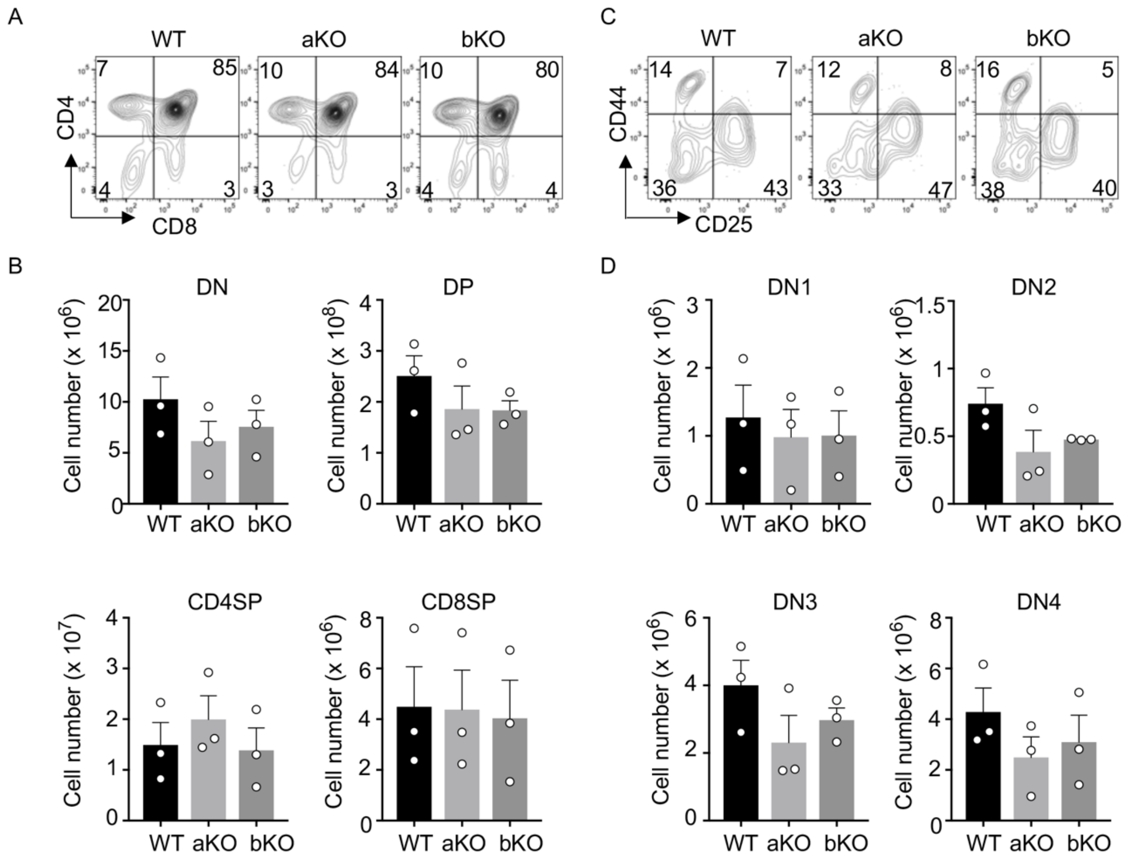

Supplementary Figure S3. T cell development in *Ccl21b*-deficient mice

(A) Flow cytometric analysis of thymocytes from 6-week-old mice. Shown are representative contour plot profiles of CD4 and CD8 expression in PI^- viable cells. Numbers in contour plots indicate the frequency of cells within the indicated area.

(B) Numbers (means and SEs; n = 3) of $CD4^-CD8^-$ (DN), $CD4^+CD8^+$ (DP), $CD4^+CD8^-TCR\beta^{high}$ (CD4SP), and $CD4^-CD8^+TCR\beta^{high}$ (CD8SP) thymocytes.

(C) Representative contour plot profiles of CD44 and CD25 expression in $CD4^-CD8^-PI^-$ viable cells.

(D) Numbers (means and SEs; n = 3) of $CD44^+CD25^-CD4^-CD8^-$ (DN1), $CD44^+CD25^+CD4^-CD8^-$ (DN2), $CD44^-CD25^+CD4^-CD8^-$ (DN3), and $CD44^-CD25^-CD4^-CD8^-$ (DN4) thymocytes.

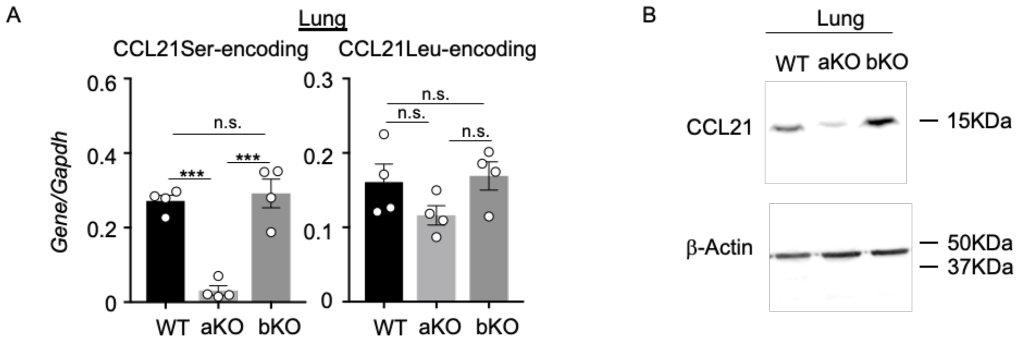

Supplementary Figure S4. CCL21 expression in the lung

(A) Quantitative RT-PCR analysis of CCL21Ser- and CCL21Leu-encoding genes (means and SEs; n = 4) in $CD45^-$ lung cells isolated from indicated mice. ***$p < 0.001$; n.s., not significant.

(B) Immunoblot analysis of CCL21 protein in the lung isolated from indicated mice. β-Actin signals were examined as loading control. Shown is a representative result from three independent experiments.

Supplementary Table S1. Primer sequences for PCR analyses

Genotyping PCR	
Primer name	Sequence
F1	5’-CTTTTTTAATTTAAAAGATCAACTC-3’
F2	5’-GTCAATAAGGAAAGACTGAGCT-3’
R1	5’-TTTATGATGTGGTAAGATCG-3’
R2	5’-GATATTGTACTGAATCTGAGGACC-3’
R3	5’-CTGCATCCCAAGATAGCATGGCTGT-3’
Quantitative genomic PCR analysis	
Primer name	Sequence
All *Ccl21* Forward	5’-CTGGTCTCATCCTCAACTCA-3’
All *Ccl21* Reverse	5’-AGGATTGCCGGGATGGGACA-3’
CCL21Ser-encoding *Ccl21a* Forward	5’-CTGGTCTCATCCTCAACTCA-3’
CCL21Ser-encoding *Ccl21a* Reverse	5’-CTTAGAGTGCTTCCGGGGTG-3’
CCL21Leu-encoding *Ccl21* Forward	5'-AAGGCAGTGATGGAGGGGGA-3'
CCL21Ser-encoding *Ccl21* Reverse	5'-GGCTTAGAGTGCTTCCGGGGTA-3’
Rag2 Forward	5’-GGCTGGCCTAAGAGATCCTG-3’
Rag2 Reverse	5’-GTTCAGGGACATCTCCTACTAAG-3’
Quantitative reverse transcription-PCR analysis	
Primer name	Sequence
CCL21Ser-encoding *Ccl21a* Forward	5’-AAGGCAGTGATGGAGGGGGT-3’
CCL21Ser-encoding *Ccl21a* Reverse	5’-CTTAGAGTGCTTCCGGGGTG-3’
CCL21Leu-encoding *Ccl21* Forward	5'-AAGGCAGTGATGGAGGGGGA-3′
CCL21Ser-encoding *Ccl21* Reverse	5'-GGCTTAGAGTGCTTCCGGGGTA-3’
Gapdh Forward	5'-CCGGTGCTGAGTATGTCGTG-3′
Gapdh Reverse	5'-CAGTCTTCTGGGTGGCAGTG-3′

Supplementary Table S2. Antibodies used in this study

	Antibody	Manufacturer	Identifier
Western blotting	Rabbit anti-mouse EXODUS-2 (CCL21)	Bio-Rad Laboratories	Cat# AAM27, RRID:AB_2072089
	Rabbit β-Actin Ab	Gene Tex	Cat# GTX109639, RRID:AB_194957
	Mouse anti-rabbit IgG-HRP	Santa Cruz Biotechnology	Cat# sc-2357, RRID:AB_628497
Immunofluorescence analysis	Rabbit anti-mouse EXODUS-2 (CCL21)	Bio-Rad Laboratories	Cat# AAM27, RRID:AB_2072089
	Alexa Fluor 647 anti-mouse Ly51 Ab	BioLegend	Cat# 108312, RRID:AB_2099613
	FITC anti-mouse CD8 Ab	BioLegend	Cat# 100706, RRID:AB_312745
	Biotin Ulex europaeus Agglutinin 1 (UEA1)	Vector Laboratories	Cat# B-1065, RRID:AB_2336766
	Goat anti-Rabbit IgG (H+L) Highly Cross-Adsorbed Secondary Antibody, Alexa Fluor™ 488	Thermo Fisher Scientific	Cat# A-11034, RRID:AB_2576217
	Streptavidin Alexa Fluor 405	Thermo Fisher Scientific	Cat# AAM27
Flow cytometric analysis	PE/Cy7 anti-mouse CD326 (EpCAM) Ab	BioLegend	RRID:AB_1236471
	eFluor450 anti-mouse CD45 Ab	Thermo Fisher Scientific	Cat# 48-0451-82, RRID:AB_1518806
	Alexa Fluor 647 anti-mouse Ly51 Ab	BioLegend	Cat# 108312, RRID:AB_2099613
	Biotin Ulex europaeus Agglutinin 1 (UEA1)	Vector Laboratories	Cat# B-1065, RRID:AB_2336766
	Ulex europaeus Agglutinin 1 (UEA1), Fluorescein	Vector Laboratories	Cat# FL-1061
	Streptavidin APC-eFluor 780	eBioscience	Cat# 47-4317-82, RRID:AB_10366688
	Rabbit anti-mouse EXODUS-2 (CCL21)	Bio-Rad Laboratories	Cat# AAM27, RRID:AB_2072089
	Rabbit anti-mouse EXODUS-2 (CCL21): Biotin	Bio-Rad Laboratories	Cat# AAM27B, RRID:AB_2072088
	Goat anti-Rabbit IgG (H+L) Highly Cross-Adsorbed Secondary Antibody, Alexa Fluor™ 488	Thermo Fisher Scientific	Cat# A-11034, RRID:AB_2576217
	APC anti-mouse CD4 Ab	Invitrogen	Cat# 17-0042-81, RRID:AB_469322
	Pacific blue anti-mouse CD8 Ab	Invitrogen	Cat# MCD0828TR, RRID:AB_2539693
	PE anti-mouse TCRb Ab	BioLegend	Cat# 109207, RRID:AB_313430
	PE/Cy7 anti-mouse CD44 Ab	Invitrogen	Cat# 25-0441-82 RRID:AB_469623
	FITC anti-mouse CD25 Ab	BioLegend	Cat# 102006, RRID:AB_312855

Western blot raw data

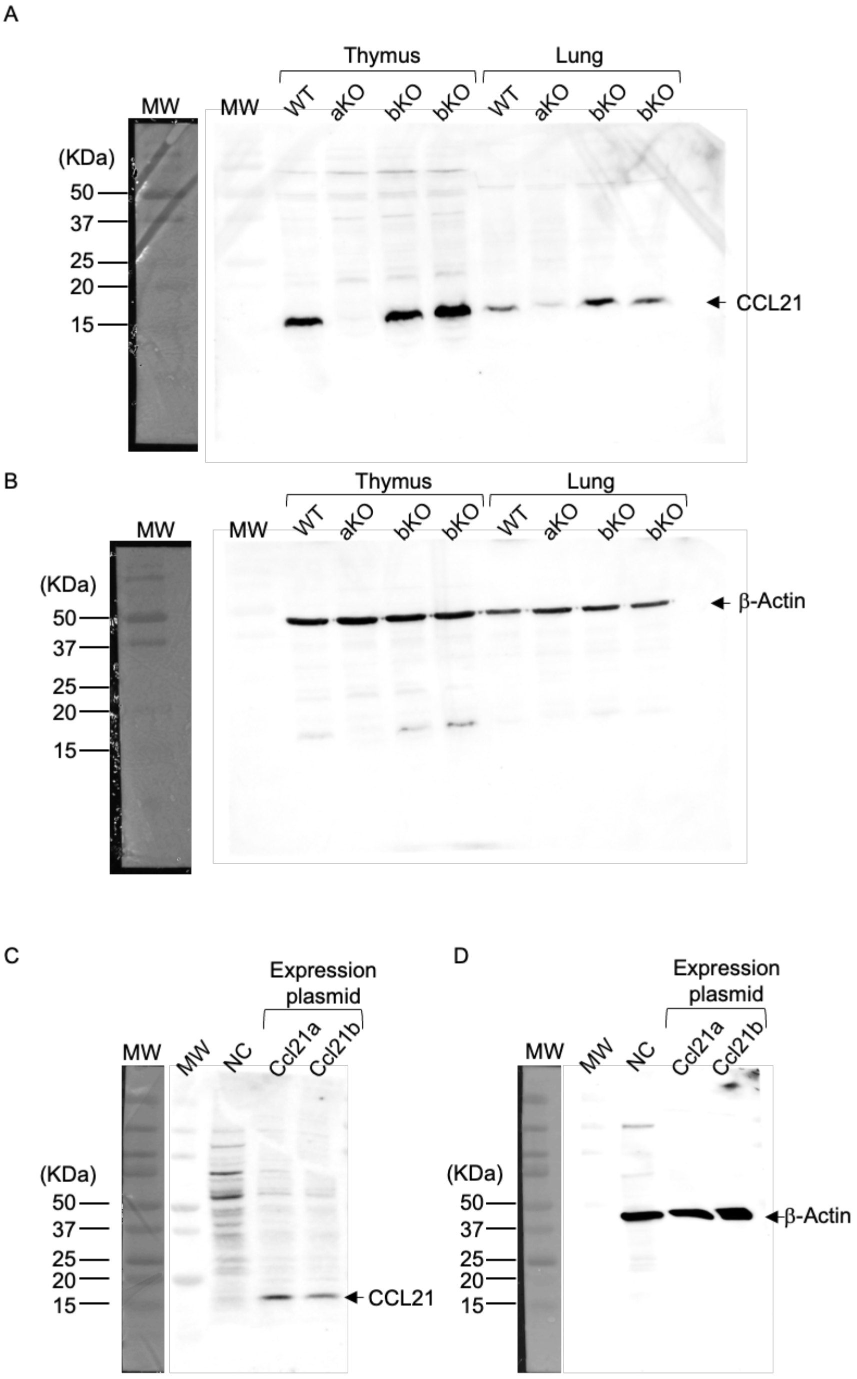

Unprocessed original images of Figures 3A and S4B (A, B) and Figure S2A (C, D). The leftmost image in each panel shows white epi-illumination. MW, molecular weight ladder marker.
